# Spinal Glutamate Transporters Are Involved in the Development of Electroacupuncture Tolerance

**DOI:** 10.3390/ijms17030357

**Published:** 2016-03-10

**Authors:** Luying Cui, Yi Ding, Jie Zeng, Yan Feng, Meng Li, Mingxing Ding

**Affiliations:** College of Veterinary Medicine, Huazhong Agricultural University, Wuhan 430070, China; dwyxcly@126.com (L.C.); yidi@webmail.hzau.edu.cn (Y.D.); strawberry25@126.com (J.Z.); fy1824914618@163.com (Y.F.); limeng_0526@163.com (M.L.)

**Keywords:** electroacupuncture tolerance, GLAST, GLT-1, EAAC1, riluzole, tail flick latency, rat

## Abstract

Background: Electroacupuncture (EA) tolerance is a gradual decline in EA antinociception because of its repeated or prolonged use. This study aims to explore the role of spinal glutamate transporters (GTs) in EA tolerance (EAT). Methods: Rats were treated with EA once per day for eight consecutive days, and their L4-5 spinal cords were collected at days 0, 2, 4, 6 and 8. The levels of three spinal GTs and their mRNAs were detected with Western blot and pPCR, respectively. Then, riluzole, a positive GT regulator, was administered intrathecally in order to observe its effect on EA analgesia after repeated EA. Results: The expression levels of the spinal GTs increased at days 2 and 4, and gradually decreased as the times of EA increased. At day 8, no difference was observed in the spinal GTs between the sham treatment and the EA treatment. Intrathecal administration of riluzole dose-dependently attenuated the decreased EA analgesia. Conclusion: These results indicated the participation of the spinal GTs in EAT.

## 1. Introduction

Acupuncture, a type of traditional Chinese therapeutic method, has been used since ancient times. It has been confirmed that electroacupuncture (EA), derived from traditional hand acupuncture, can relieve various types of pain with few side effects [1,2]. However, this analgesic effect of EA will become attenuated and finally disappear after prolonged or repeated EA stimulations, which is termed “EA tolerance” (EAT) [3].

A great deal of attention has been paid by practitioners and researchers to EAT. Studies have verified that EA induced antinociception, mainly via the release of endogenous opioids peptides [4,5], which act on their receptors. In studies of EAT, decreased numbers of opioid receptors after repeated EA have been reported, and possibly contributes to declined EA analgesia [6,7]. Moreover, EA induced the release of anti-opioid peptides, such as cholecystokinin octapeptide, orphanin FQ and angiotensin II, and also attenuates EA analgesia and contributes to EAT [8]. Other substances, such as N-methyl-d-aspartic acid receptor (NMDAR) [9], 5-HT [3,10], norepinephrine [11], and Ca^2+^ [12,13], have been demonstrated to be correlated with EAT. However, the molecular mechanism of EAT is controversial.

It has been demonstrated that the inhibition of NMDAR can enhance acupuncture antinociception and delay EAT development [9,14]. The homeostasis of the extrasynaptic glutamate, a main ligand of NMDAR, is maintained by glutamate transporters (GTs) [15]. Among the identified GTs, l-glutamate-l-aspartate transporter (GLAST), glutamate transporter 1 (GLT-1) and excitatory amino-acid carrier 1 (EAAC1) are widely distributed in the central nervous system [16]. GLAST and GLT-1 mainly exists in glial cells, whereas EAAC1 is basically in neurons [17]. Evidence has shown the involvement of GTs in EA analgesia. Kim *et al.* reported that EA induced an increase in glial transporter expression, with the paw withdrawal latency increased in complete Freund’s adjuvant (CFA)-treated rats [2]. However, no study has been reported regarding the role of GTs in EAT.

The inhibition of glutamate transporter activity directly contributes to a heightened activity of NMDAR and the development of opioid tolerance [18]. Rats tolerant to morphine showed decreased protein expression of the three spinal glutamate transporters [18,19]. Combined administration of morphine with MS-153 (an activator of GLT-1) [20] or riluzole (a positive GT regulator) [19] attenuated, but with l-*trans*-pyrrolidine-2,4-dicarboxylate (a GT inhibitor) potentiated, the development of morphine tolerance. It has been demonstrated by Han *et al.* that there was a bi-directional cross-tolerance between EA and morphine [21], implying a possible involvement of spinal GTs in EAT.

Here, rats were treated with EA, once per day for eight days, to set up an EAT model. The dynamic expression profiles of spinal GTs were characterized at both mRNA and protein levels before (day 0) and at days 2, 4, 6, and 8 of EA. The involvement of GTs in EAT was further confirmed through intrathecal administration (i.t.) of riluzole, a positive GT regulator. Our results showed that the spinal GTs were downregulated after repeated EA, which, in turn, contributes to the EAT development.

## 2. Results

### 2.1. Dynamic Changes in Tail Flick Latency of Rats with Repeated EA

**Figure 1:**
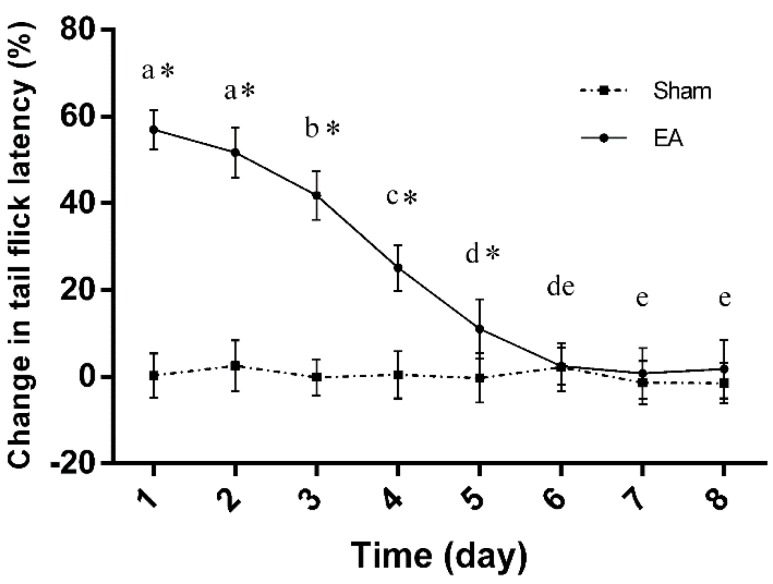
Changes in tail flick latency after repeated electroacupuncture (EA) (Mean ± SD, %, *n* = 6). EA was given once daily for eight consecutive days. Rats in the sham group were treated with needles left on the acupoints, but without electricity. The significance of differences was calculated by a *t*-test or a one-way ANOVA. The values with different letters (a–e) within the EA treatment differ (*p* < 0.05); * *p* < 0.05 compared with the sham group within the same day.

Tail flick latency (TFL) was measured every day throughout the experiment (Figure 1). No differences (*p* > 0.05) were found in TFL in the sham treatment during this trial. Compared with the sham treatment, the alteration of TFL in EA treatment was higher (*p* < 0.05) from day 1 to day 5, but did not differ (*p* > 0.05) from the sham treatment from day 6 to day 8. In EA treatment, the TFL percentage alteration was 59.6% ± 4.6% at day 1, then declined (*p* < 0.05) to 41.8% ± 5.6% at day 3, and fell (*p* < 0.05) to 1.9% ± 7.4% at day 8, implying EAT formation.

### 2.2. Expression of Spinal GTs Induced by Single and Repeated EA in Rats

**Figure 2:**
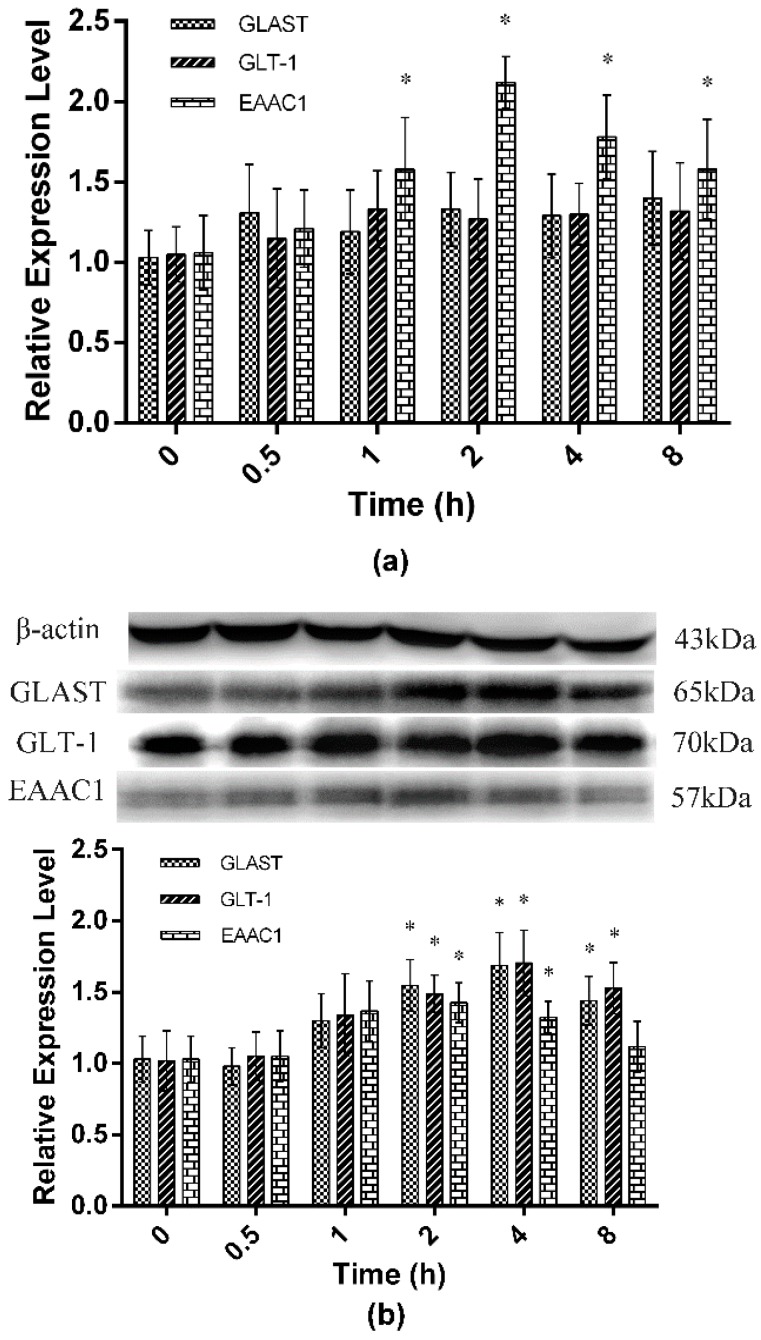
The expressions of the spinal GTs after single EA (Mean ± SD, *n* = 6). Samples were collected from the L4-5 segment of spinal cord. (**a**) MRNA expression; (**b**) protein expression. The significance of differences was calculated by a *t*-test. * *p* < 0.05 compared with 0 h within the same group.

The time-course expression of spinal GTs induced by single EA were determined to select the optimal sampling time points for repeated EA experiments (Figure 2). The results showed that single EA induced no change (*p* > 0.05) in GLAST and GLT-1 mRNA expressions, but an increase in EAAC1 mRNA expression (*p* < 0.05) from 1 to 8 h, with the peak at 2 h (*p* < 0.05). Both GLAST and GLT-1 levels increased (*p* < 0.05) from 2 to 8 h, with their peak levels at 4 h, whereas EAAC1 level increased (*p* < 0.05) from 2 to 4 h, with the peak at 2 h after single EA. Therefore, for the experiment of repeated EA, the samples for spinal GLAST and GLT-1 expressions were taken at 4 h while samples for spinal EAAC1 expression were taken at 2 h, after each EA.

**Figure 3:**
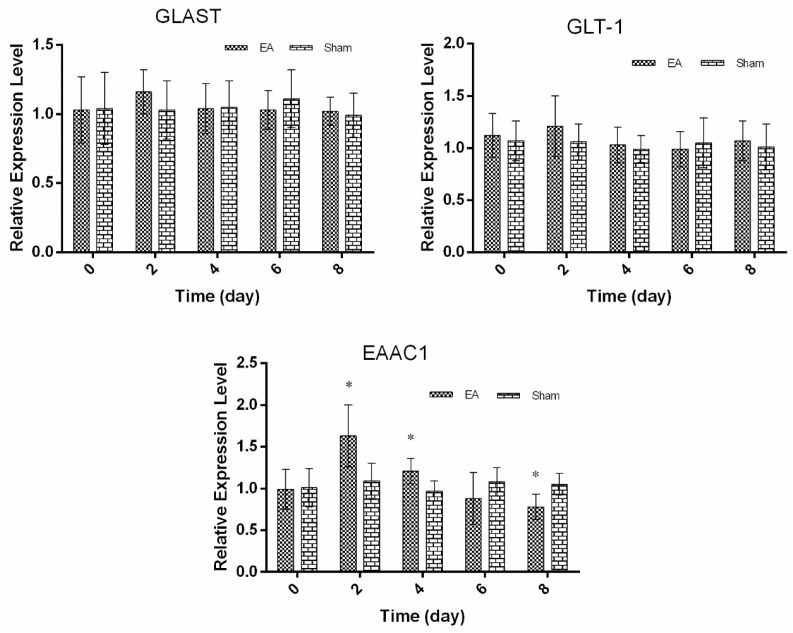
The protein expression levels of the spinal GTs after single EA (Mean ± SD, *n* = 6). Samples were collected from the L4-5 segment of spinal cord. The significance of differences was calculated by a *t*-test. * *p* < 0.05 compared with 0 h within the same group.

**Figure 4:**
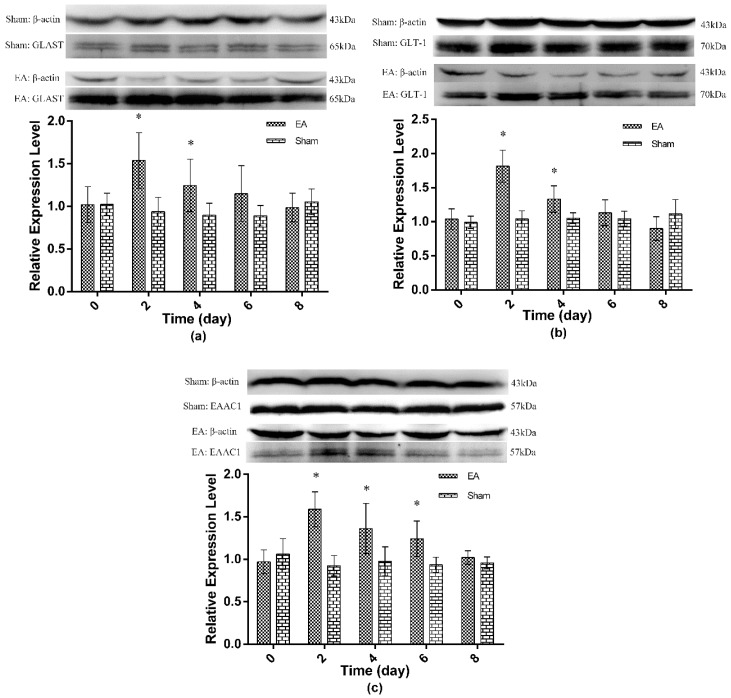
The protein expression levels of the spinal GTs after repeated EA (Mean ± SD, *n* = 6). Rats were given EA once daily for eight consecutive days. Rats in the sham group were treated the same as EA group but without electricity. Samples were collected from the L4-5 segment of the spinal cord at 2 h (for EAAC1) or 4 h (for GLAST and GLT-1) immediately after the last EA or sham treatment. (**a**) GLAST expression; (**b**) GLT-1 expression; (**c**) EAAC1 expression. The significance of differences was calculated by a *t*-test. * *p* < 0.05 compared with sham group within the same day.

For the repeated EA experiments, the expression profiles of spinal GTs were determined to investigate their involvement in EAT. The results showed no change in the expression of spinal GTs in the sham treatment after repeated EA (Figure 3 and Figure 4). Compared with the sham treatment, mRNA expressions of GLAST and GLT-1 were unchanged (*p* > 0.05) in the EA treatment during the experiments. EA caused EAAC1 mRNA expression to increase (*p* < 0.05) at day 2 and day 4, but to decrease at day 8 (*p* < 0.05) compared with the sham treatment (Figure 3). The levels of the three GTs in EA treatment shared a similar pattern; EA induced GLAST and GLT-1 to increase (*p* < 0.05) at days 2 and 4, and EAAC1 to increase at days 2 to 6, but the three GTs to approximate (*p* > 0.05) to those in the sham treatment at day 8. Statistical analysis showed that the TFL change had a positive correlation with GLAST (*r* = 0.513, *p* < 0.05), GLT-1 (*r* = 0.791, *p* < 0.05) and EAAC1 (*r* = 0.549, *p* < 0.05) levels (Figure 4).

### 2.3. The Effect of Riluzole on Changes in Tail Flick Latency Induced by Repeated EA

**Figure 5:**
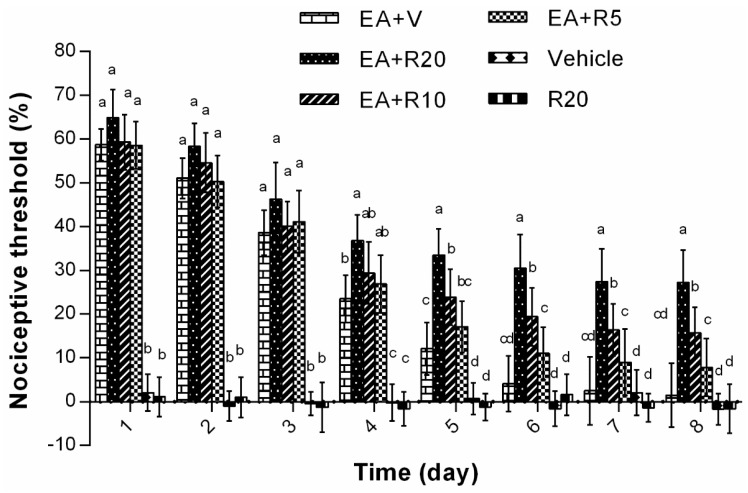
The effect of intrathecal administration of riluzole on changes of tail flick latency in EA-treated rats (Mean ± SD, %, *n* = 6). EA was given once per day for eight consecutive days. Riluzole or vehicle was given once daily 30 min prior to EA. The significance of differences was calculated by a one-way ANOVA. EA + V: EA plus vehicle; EA + R20: EA plus 20 μg riluzole; EA + R10: EA plus 10 μg riluzole; EA + R5: EA plus 5 μg riluzole; R20, 20 μg riluzole. The values with different letters (a–d) at the same day differ (*p* < 0.05).

The effect of i.t. riluzole on EAT formation was investigated. As shown in Figure 5, no differences (*p* > 0.05) were observed in TFL alteration in the vehicle treatment and in 20 μg riluzole (R20) treatment during the experiments. In the groups of EA plus vehicle (EA + V), EA plus 5 μg riluzole (EA + R5), EA plus 10 μg riluzole (EA + R10), and EA plus 20 μg riluzole (EA + R20), the percentage changes in TFL decreased (*p* < 0.05) as the times of EA increased. In EA + V and EA + R20, the alterations in TFL decreased from 58.7% ± 3.6% to 64.9% ± 6.4% at day 1 to 1.5% ± 7.3% and 27.2% ± 7.4% at day 8, respectively. Compared with EA + V, the TFL alterations were greater (*p* < 0.05) in EA + R20 from days 4 to 8, and in EA + R10 from days 5 to 8. No differences (*p* > 0.05) were observed between EA + R5 and EA + V group during this trial.

## 3. Discussion

Our pre-experiment showed no differences in TFL between the sham treatment and the control treatment (the rats were only restrained in the cylinder). Such results indicated that there was no non-specific physiological effect to needle insertion. Cheng *et al.* studied dynamic changes of EA analgesia, and the expression patterns of opioid peptides and their corresponding receptors, and found no differences in pain threshold, and expression levels of these opioids and receptors in the analgesia-related brain nuclei or areas between the sham treatment and the control treatment [22,23]. In EA-related studies, either the sham group or the control group is commonly chosen to compare with the test group. Therefore, in the present study, the control treatment was replaced by the sham treatment for the detection of the expression levels of the spinal GTs.

It has been verified that the EA antinociceptive effect varied markedly among individuals in both humans and rats [1,24,25]. Rats can be divided into responders and non-responders, according to percentage alterations in TFL after acupuncture. The standard of classification, such as 30% [24], 50% [26], or 60% [27], varies in different studies. Ko *et al.* determined the responders according to a significant increase in TFL [1]. In the present study, we defined rats with 50% increase in TFL as responders. The analgesic effect induced by EA has been reported by many studies. Han *et al.* found that 2 Hz EA induced an increase in TFL by 135% ± 8% [21]. Wang *et al.* reported an increase in TFL by 60.6% ± 6% in rats treated using 2 Hz EA [28]. Our study showed that the TFL alteration after 2 Hz EA was 59.5% ± 4.5%, which was similar to the report from Wang *et al.* There have been reports on acupuncture tolerance in clinical therapy [29]. It has been shown that EAT could be induced by EA on rats, once per day for 6 days [27]. In the present study, the antinociceptive effect induced by repeated EA gradually declined, and became almost ineffective at day 7, which indicated EAT formation.

Because the optimal sampling time for detecting spinal GTs expression levels after EA has not been reported, the time-course expressions of the three spinal GTs, following single EA stimulation, were determined in the present study. The samples for EAAC1 and for GLAST and GLT-1 were collected at 2 and 4 h, respectively, according to the time points at which their peak levels occurred. It has been reported by Kim *et al.* that no change was found in spinal GLAST and GLT-1 protein expressions after EA in healthy rats [2], which seems different from our results. Because the specific time point of sample collection was not mentioned in that paper, such discrepancy was probably due to the different time points of sample collection.

Glutamate uptake is essential to the maintenance of normal nociception transmission [16]. Kim *et al.* have demonstrated that EA confers an antinociceptive effect through reversing the decreased spinal GLAST and GLT-1 in CFA-injected rats [2]. In the present study, EA induced an increase in TFL and the upregulation of spinal GTs, which suggested a possible role of spinal GTs, contributory to EA analgesia. Conversely, Liaw *et al.* found that i.t. dl-threo-β-benzyloxyaspartate, a non-selective GT blocker, caused spontaneous nociceptive behaviors dose-dependently, and marked hypersensitivity when the rats were treated with thermal or mechanical stimulation [30]. Our experiments showed that repeated EA caused a gradual decline of spinal GT levels, which was positively correlated with the changes in TFL, thereby indicating the participation of spinal GTs in EAT. The mechanism of spinal GT in mediating EAT has not been reported. The similarity between EA and morphine tolerances has been demonstrated in a cross-tolerance study [21]. In morphine tolerance rats, the morphine-induced downregulation of spinal GTs were proposed to result in excitatory amino acid receptor activation through increased availability of glutamate in the synaptic space [19]. However, whether the same mechanism could provide an explanation of EAT still needs further investigation. It is noteworthy that the mRNA levels of GLAST or GLT-1 did not change in spite of their increased protein expressions in our experiment. It has been suggested that the ubiquitin-proteasome system mediated GT degradation, contributing to the GT turnover [31]. Kim *et al.* found that EA and MG-132, a proteasome inhibitor, both antagonized CFA-induced spinal GLAST and GLT-1 downregulation, suggesting that EA probably mediates GT regulation through the ubiquitin-proteasome pathway in inflammation pain [2]. In addition, palmitoylation and miR-124 have been proven to participate in the regulation of GLT-1 [32]. Therefore, in our study, the inconsistent changes between mRNA and protein levels of GLAST and GLT-1 after EA treatment were probably involved in posttranscriptional regulation. However, more investigations should be done.

Riluzole, a non-selective positive regulator of GT activity, can increase glutamate uptake both *in vivo* and *in vitro* [33,34,35]. In the present study, rats treated with i.t. riluzole at a dose of 20 μg for eight consecutive days showed no change in basal TFL (bTFL), which was consistent with previous reports [19,36]. We observed that riluzole dose-dependently attenuated the EAT development, thereby suggesting that enhanced activity of spinal GTs was, at least in part, able to compensate for the GT downregulation caused by repeated EA. This was consistent with the results from our Western blot analysis, that the levels of spinals GTs were positively correlated with the changes in TFL after repeated EA, thereby suggesting the involvement of spinal GTs in EAT.

## 4. Materials and Methods

### 4.1. Ethics Statement

The trial was approved (HZAURA-2015; 5 January 2015) by the Animal Care and Use Committee of Huazhong Agricultural University. All experimental procedures were conducted according to the guidelines of the National Institutes of Health Guide for the Care and Use of Laboratory Animals.

### 4.2. Animals

Male Sprague-Dawley rats (250 ± 20 g) were purchased from the Hubei Provincial Center for Disease Control and Prevention. The rats were fed, six to a cage, and were allowed to eat and drink freely. Before the experiments, the rats were permitted to adapt to their surroundings for 7 days.

### 4.3. Experiment Design

To select the optimal sampling time points for repeated EA, the time-course expression of spinal GTs induced by single EA was determined. Six rats were euthanized before (0 h) and at 0.5, 1, 2, 4 and 8 h, respectively, after single EA. The time points where the highest expression levels of spinal GTs appeared were determined as the optimal sampling points.

To determine the expression patterns of the spinal GTs in rats treated with repeated EA, 120 rats were randomly classified into two treatments, *i.e.*, EA treatment and sham treatment with sixty rats per treatment. The rats were treated with EA once per day for 8 days consecutively. The rats in the sham group were treated the same as the rats in EA group, but without electricity. TFL was measured every day immediately before and after EA, respectively, and the changes in TFL were calculated. Twelve rats from each group were euthanized at days 0, 2, 4, 6 and 8, respectively. Since the optimal sampling time points after single EA were 2 h for EAAC1 and 4 h for GLAST and GLT-1, six out of the twelve rats were euthanized at 2 or 4 h after EA or sham treatment.

To further explore the involvement of spinal GTs in EAT, riluzole, a non-selective positive GT regulator, was intrathecally administered. Thirty-six rats were allotted to six groups: EA + V group, EA + R5 group, EA + R10 group, EA + R20 group, vehicle group and R20 group. All rats underwent intrathecal placement surgery. EA was given once per day for 8 days, consecutively. The doses of riluzole were applied according to previous studies [19]. Riluzole or vehicle was given once daily. For rats treated with EA, the i.t. injection was given 30 min before EA. The TFL was examined every day, immediately before and after the injection (for R20 and vehicle groups) or after EA (for EA + riluzole groups and EA + vehicle group), respectively.

### 4.4. EA Stimulation

EA was applied at 9:00 a.m. with an ambient temperature of 21–23 °C, and was manipulated according to a previous report [37]. The rats were restrained in a polyethylene cylinder without anesthesia. Their hind legs and tails were free. Before the experiment, they were accustomed to the cylinder for three days. The skin of the hind legs was sterilized with 75% alcohol. The bilateral acupoints “Zusanli” and “Sanyinjiao” were selected for the present study. The “Zusanli” (ST36) acupoints were located 4 mm laterally to the anterior tuberpoint of the tibia. The “Sanyinjiao” (SP6) acupoints were located 3 mm proximally to the medial malleolus at the distal tibia. The acupuncture needle, with a diameter of 0.30 mm and a length of 13 mm, was pricked into the “Zusanli” or “Sanyinjiao” acupoints to a depth of 7 or 5 mm, respectively. Then the needles were connected to the Acupunctoscope (Xindonghua Electronic Instrument Co., Ltd., Beijing, China), which was set at square waves. The EAs were 3 mA in amplitude and 2 Hz in frequency, and lasted for 30 min.

### 4.5. Assay of Tail Flick Latency and Selection of Responders

The nociceptive threshold was determined by measuring the TFL [37]. Briefly, the radiant heat was applied to the proximal third of the tail. The duration time from the exposure of the heat to the tail flick response was recorded. The basal latency was controlled at 4–6 s by adjusting the power of the thermal stimulus (ZS Dichuang Science and Technology Development Co., Ltd., Beijing, China). A cut-off limit of 15 s was set to prevent tail injury. The TFL was recorded before and immediately after EA. At each time point, the TFL was determined 3 times with 5 min intervals. The percentage change of TFL was determined as: TFL (%) = (TFL after EA − bTFL)/bTFL × 100%.

The rats that showed more than a 50% increase in TFL were selected for the present study. The experiments started 7 days after the selection.

### 4.6. RT-PCR

The segment L4-5 of the spinal cord was obtained and was ground in liquid nitrogen. RNA was isolated using Trizol reagent (Invitrogen, Carlsbad, CA, USA) and was reverse-transcripted using a First Strand cDNA Synthesis Kit (TOYOBO, Osaka, Japan). The qPCR was performed according to the manufacturer’s protocol (Applied Biosystems, Carlsbad, CA, USA). The primer sequences of GTs are shown in Table 1. Rat *gapdh* primers (Sangon Biotech Co., Ltd., Shanghai, China) were used as an internal control. The mRNA of GLAST, GLT-1 and EAAC1 relative to *gapdh* mRNA were quantified with the 2^−^^Δ*C*t^ method, where Δ*C*_t_ = *C*_t target gene_ − *C*_t *gapdh*_.

### 4.7. Western Blot

Proteins were extracted from the grounded spinal cord segment using RIPA Lysate Buffer according to the manufacturer’s instruction (Beyotime Biotech, Nantong, China). The crude protein per sample was subjected to 10% SDA-PAGE and Western blotted on PVDF membrane using the Mini-PROTEIN Tetra Cell (Bio-Rad, Hercules, CA, USA). The membrane was incubated in blocking buffer with 5% nonfat milk for 2 h, and was subsequently immunolabeled overnight at 4 °C with rabbit anti-GLAST (1:500), anti-GLT-1 (1:500), anti-EAAC1 (1:500) or anti-β-actin (1:300, Santa Cruz Biotechnology, Santa Cruz, CA, USA), respectively. The membrane was washed and treated with horseradish-peroxidase-conjugated anti-rabbit secondary antibody (1:5000, Boster biotech, Wuhan, China) for 1 h at 4 °C. Visualization of the antigen-antibody complex was conducted with a horseradish peroxidase Substrate (Millipore, Billerica, MA, USA) using the ImageQuant LAS 4000 min CCD camera (GE Healthcare, Piscataway Township, NJ, USA). The bands were analyzed by Quantity One software (Bio-Rad). Beta-actin was used as the internal control. Values of GT were represented as the ratio of the optical density of the GT band to the density of the related β-actin band.

### 4.8. Intrathecal Placement

The rats were deprived of food overnight before surgery and were intraperitoneally injected with sodium pentobarbital (30 mg/kg). A stretched polyethylene (PE)-10 catheter (Smith Medical, Dublin, OH, USA) was inserted at L5-6 intervertebral space through a 20-gauge guide-cannula (0.9 × 35 mm) and advanced about 3 cm beyond the tip of cannula to the lumbar enlargement of the spinal cord, with the other end of the catheter tunneled through the skin in the occipital region. The full length of catheter was about 18 cm with the dead space controlled at less than 15 μL. After catheterization the animals were kept in individual cages and permitted recovery from the operation for 7 days before testing. Those having neurological symptoms after operation were excluded from the trial. Riluzole (Selleckchem, Houston, TX, USA) was dissolved in DMSO (0, 5, 10 or 20 mM for each group, respectively) and diluted in saline to the needed dosages. Then, the riluzole solution was intrathecally administered by inserting a micro syringe needle (Gaoge, Shanghai, China) into the catheter with 15 μL, followed by a 20-μL saline flush, at a rate of 10 μL/min.

### 4.9. Statistical Analysis

Data were analyzed with SPSS 21.0 (IBM Co., Armonk, NY, USA). All data are shown as mean ± SD. Independent *t*-test was used to analyze variables, including protein and mRNA expression levels, and the TFL alteration between the EA treatment and sham treatment. Data including TFL change after repeated EA, and the TFL change among the groups with different doses of riluzole, were analyzed with One-way ANOVA. Bonferroni test was used when significant differences were found. The Pearson’s method was used to detect the correlations between the alterations in TFL and levels of spinal GTs. *p* < 0.05 was considered significant.

## 5. Conclusions

Our study established chronic EAT model in rats by providing EA once per day for eight days. Repeated EA induced down-regulation of the spinal GTs at the protein level. i.t. riluzole, a positive GT regulator, attenuated EAT. These results indicated the participation of the spinal GTs in EAT development.

## Figures and Tables

**Table 1:** Primer sequences of glutamate transporters.

Name	Accession Number	Primer Sequence
*GLAST*	NM_019225.2	F: 5′-TATACAGTGACAGTCATCGTC-3′
		R: 5′-ACAAATCTGGTGATGCGT-3′
*GLT-1*	NM_017215.2	F: 5′-TCCTGGATGGAGGTCAGATAG-3′
		R: 5′-CTCGGACTTGGAAAGGTGATAG-3′
*EAAC1*	NM_013032.3	F: 5′-TACTGGGCATCGTGGTAGGA-3′
		R: 5′-TACAGCAATGACGGTGGTGG-3′

## Abbreviations

EA	Electroacupuncture
GT	Glutamate transporter
GLAST	l-Glutamate-l-aspartate transporter
GLT-1	Glutamate transporter 1
EAAC1	Excitatory amino-acid carrier 1
NMDAR	*N*-Methyl-d-aspartic acid receptor
CFA	Complete Freund’s adjuvant
i.t.	Intrathecal administration
TFL	Tail flick latency
bTFL	Basal tail flick latency
